# Environmental pollutants, a possible etiology for premature ovarian insufficiency: a narrative review of animal and human data

**DOI:** 10.1186/s12940-017-0242-4

**Published:** 2017-04-07

**Authors:** Pauline Vabre, Nicolas Gatimel, Jessika Moreau, Véronique Gayrard, Nicole Picard-Hagen, Jean Parinaud, Roger D. Leandri

**Affiliations:** 1 Médecine de la Reproduction, CHU de Toulouse, Hôpital Paule de Viguier, 330 avenue de Grande Bretagne, F-31059 Toulouse Cedex, France; 2 Université de Toulouse; UPS; Groupe de Recherche en Fertilité Humaine (EA 3694, Human Fertility Research Group), F-31059 Toulouse, France; 3 Institut National de Recherche Agronomique, Unité Mixte de Recherche 1331, Toxalim, Research Center in Food Toxicology, F-31027 Toulouse, France; 4 Université de Toulouse, Institut National Polytechnique de Toulouse, Ecole Nationale Vétérinaire de Toulouse, Ecole d’Ingénieurs de Purpan, Université Paul Sabatier, F-31076 Toulouse, France

**Keywords:** Premature ovarian insufficiency, Ovarian reserve, Environment, Pollutants, Fertility

## Abstract

**Background:**

Because only 25% of cases of premature ovarian insufficiency (POI) have a known etiology, the aim of this review was to summarize the associations and mechanisms of the impact of the environment on this pathology.

**Main body of the abstract:**

Eligible studies were selected from an electronic literature search from the PUBMED database from January 2000 to February 2016 and associated references in published studies. Search terms included ovary, follicle, oocyte, endocrine disruptor, environmental exposure, occupational exposure, environmental contaminant, pesticide, polyaromatic hydrocarbon, polychlorinated biphenyl PCB, phenol, bisphenol, flame retardant, phthalate, dioxin, phytoestrogen, tobacco, smoke, cigarette, cosmetic, xenobiotic. The literature search was conducted in accordance with the Preferred Reporting Items for Systematic Reviews and Meta-Analyses (PRISMA) guidelines. We have included the human and animal studies corresponding to the terms and published in English. We have excluded articles that included results that did not concern ovarian pathology and those focused on ovarian cancer, polycystic ovary syndrome, endometriosis or precocious puberty. We have also excluded genetic, auto-immune or iatrogenic causes from our analysis. Finally, we have excluded animal data that does not concern mammals and studies based on results from in vitro culture. Data have been grouped according to the studied pollutants in order to synthetize their impact on follicular development and follicular atresia and the molecular pathways involved.

Ninety-seven studies appeared to be eligible and were included in the present study, even though few directly address POI. Phthalates, bisphenol A, pesticides and tobacco were the most reported substances having a negative impact on ovarian function with an increased follicular depletion leading to an earlier age of menopause onset. These effects were found when exposure occured at different times throughout the lifetime from the prenatal to the adult period, possibly due to different mechanisms. The main mechanism seemed to be an increase in atresia of pre-antral follicles.

**Conclusion:**

Environmental pollutants are probably a cause of POI. Health officials and the general public must be aware of this environmental effect in order to implement individual and global preventive actions.

## Background

Over the last few decades, much evidence has been gathered that confirms that certain chemical, physical and biological substances present in our environment produce harmful effects on human reproduction [1].

Environmental pollutants refer to all of the exogenic, non-essential factors for humans, which, when released into the environment, can be detrimental to human health and/or to the environment. The industrialization of our society maximizes the use of these substances and poses a real problem for public health [2–6].

Premature ovarian insufficiency (POI) is a medical and biological diagnosis that affects young women, altering their quality of life and their fertility. According to the European Society of Human Reproduction and Embryology (ESHRE) guidelines [7], POI is defined by oligo/amenorrhea for at least 4 months and an elevated FSH level > 25 IU/l on two occasions > 4 weeks apart. Its prevalence is about 1% in women before the age of 40 [7]. Despite this precise definition, this medical diagnosis is very often confused with two other diagnoses that share many pathophysiological substrates: premature menopause (before age 45) for which POI is a major risk factor, and diminished ovarian reserve, which can be a one of the warning signs of POI [8]. The ESHRE Guideline Group on POI defines ovarian reserve [7] as “encompassing both the quantity and quality of primordial follicles”.

**Fig. 1:**
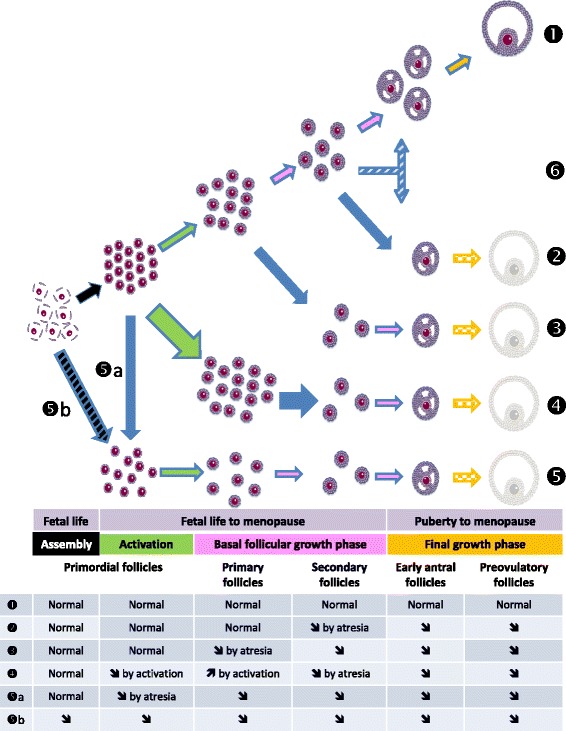
The different mechanisms inducing POI. Folliculogenesis (❶) begins after assembly (*black arrows*) of primordial follicles during second trimester of pregnancy. Activation of primordial follicles (*green color*) to enter the growth phase of folliculogenesis is continuous from third trimester of fetal life to menopause. This activation is driven by local factors and is independent of gonadotropins. Basal follicular growth (*pink color*) is driven by paracrine factors. From the early antral follicle stage, their growth depends on gonadotrophins to enter the final phase (*orange color*) up to ovulation. From the beginning, follicles undergo physiological atresia (*blue lines around arrows*), that participate to the decrease of the pool with years. Mechanisms leading to POI (symbolized by a final pale preovulatory follicle,): • decrease of the pool of primordial follicles (❺) either due to massive atresia (❺a) or default in assembly (❺b). • increase in follicular apoptosis concerning any other follicular stage (❷ and ❸). • increase of the activation (*large green arrow*) of the resting pool of primordial follicles (❹) resulting in its exhaustion. It is generally followed by increased atresia at following follicular stages (*large blue arrow*). • default in basal follicular growth leading to its arrest before the antral stages preventing ovulation while the pool of primordial follicles is normal (❻)

Etiologies of POI are still poorly defined because in more than 75% of cases, the cause is undetermined [9, 10]. Genetic [11], iatrogenic [12], immunologic [13], metabolic [14] and infectious [15, 16] causes have been reported (Table 1). Four main mechanisms can lead to POI (Fig. 1): i) exhaustion of the pool of resting primordial follicles (either constitutive due to default in their assembly, or acquired because of their increased atresia), ii) increased follicular atresia, iii) an increased activation of primordial follicles and iv) a blockage of folliculogenesis before the antral stages preventing ovulation.

**Table 1:** Main etiologies of premature ovarian insufficiency

Etiologies	Characteristics
Genetics: [11] - Chromosomal abnormality - Genetic mutations	- Monosomy X and mosaicism: Turner syndrome, triploid syndrome, partial deletion and X translocation - X chromosomes: FMR1 premutation: Fragile X syndrome - Other chromosomes: FOX L2 (blepharophimosis, ptosis), FSHR, NOBOX, GALT, SF1, GDF1, etc.
Iatrogenic: [12]	- Pelvic, ovarian surgery - Chemotherapy: alkylating agents ++, etc. - Radiotherapy
Autoimmune disease: [13]	- Isolated - Associated: hypothyroidism, type 1 diabetes, Addison’s disease, myasthenia, lupus, etc.
Metabolic: [14]	- Congenital galactosemia - 17-hydroxylase deficiency
Infectious: [15, 16]	- HIV infection - Mumps

Environmental factors seem to be major determinants in the ovarian reserve or in premature menopause acting during the prenatal period or during the adult life [17]. Because of this, they are suspected of contributing to the onset of POI. Although the possible environmental impact on POI is often alluded to, the evidence for this relationship has yet not been evaluated.

Environmental pollutants can impact ovarian function in three ways that can coexist:

- ***Endocrine disrupting chemicals (EDCs)*** *:* EDCs have been defined by the Endocine Society as “an exogenous chemical, or mixture of chemicals, that interferes with any aspect of hormone action”. Depending on the time of exposure regarding ovarian ontogenesis, EDCs’ effects on ovarian functions can be transitory or permanent. They can influence ovarian reserve by acting mainly on the aryl hydrocarbon receptor (AhR) or estrogen receptors (ERs). After binding to its exogenous ligand, AhR translocates toward the nucleus, it associates with a nuclear receptor and is able to bind to DNA sequences and modulates them. AhR induces a Bax synthesis, which is a pro-apoptotic factor contributing to follicular atresia [18]. ERs play evident roles during the gonadotrophins dependant phase of folliculogenesis. However arguments for their role from the very early phases of folliculogenesis are given by the fact that they are increasingly expressed from the primordial stage onward in adult human follicles [19] and consistently expressed by oocytes in human fetal ovaries whatever the follicular stage [20]. In different mammals, estrogens are known to interfere with primordial follicles formation: in a positive manner in primates and bovines, in a negative manner in mice [20].
- ***Induction of oxidative stress***: This occurs when cell mechanisms regulating the level of reactive oxygen species (ROS) are overwhelmed and responsible for an imbalance of ROS. The accumulation of ROS can harm ovarian function. There is solid proof that ROS, induced by environmental factors, are involved in the initiation of antral follicle apoptosis [21]. It was also highlighted that the anti-oxidant capacities differed depending on the follicle stage. A cross-sectional study confirmed the role of oxidative stress in POI pathogenesis by showing that the oxidative stress markers were increased in patients with POI compared to a control group [22].
- ***Epigenetic modifications***: Exposure to environmental pollutants led to modifications in DNA methylation altering ovarian function and, if these modifications affect the germline in a stable way, it will promote transgenerational inheritance of altered ovarian function [23–25].

We wanted to study the role that the environmental contaminants play in the occurrence of POI by conducting a systematic review of the scientific literature, which includes human and animal data.

## Methods

### Research strategy

We have conducted a review of the literature concerning the exposure to environmental pollutants and POI. Regarding the articles selected from the literature, our selection was made in compliance with the PRISMA (Preferred Reporting Items for Systematic Reviews and Meta-Analyses) criteria [26].

We conducted the research in February 2016 using the PubMed database. All of the research was done using the Advanced Search Builder and the key words were searched in [Title OR Abstract]. We limited the search to the period from 01/01/2000 to 02/26/2016 and to articles written in English.

Our search strategy is summarized in Table 2. Regarding the ovary, we used comprehensive terms in order to optimize the search. Regarding the environment, we opted for a two prong strategy that, in the first stage, combined comprehensive terms and in the second stage, specified by the pollutant names so as not to omit any articles.

**Table 2:** Search strategy: list of keywords used

Search 1		
Ovary OR Ovaries OR Ovarian OR Oocyte* OR Ovocyte* OR Follicle* OR Follicular	AND	Endocrine disruptor* OR Environmental exposure* OR Occupational exposure* OR Environmental contaminant
Search 2		
Ovary OR Ovaries OR Ovarian OR Oocyte* OR Ovocyte* OR Follicle* OR Follicular	AND	Pesticide* OR Polyaromatic hydrocarbon* OR Polychlorinated biphenyl* OR PCB* OR Phenol*OR Bisphenol* OR Flame retardant* OR Phthalate* OR Dioxin* OR Phytoestrogen* OR Tobacco OR smoke* OR cigarette* OR Cosmetic* OR Xenobiotic

In all, we have combined the results from search 1 with those from search 2 according to the following algorithm: Search 1 OR Search 2.

### Article inclusion and exclusion criteria

We have included all of the human and animal studies and reviews responding to the subjects of our search.

We have excluded articles that included results that did not concern ovarian pathology, those focusing on ovarian cancer, polycystic ovary syndrome, endometriosis or precocious puberty. We have also excluded genetic, auto-immune or iatrogenic causes from our analysis. Finally, we have excluded animal data that does not concern mammals and studies based on results from in vitro culture.

## Results

**Fig. 2:**
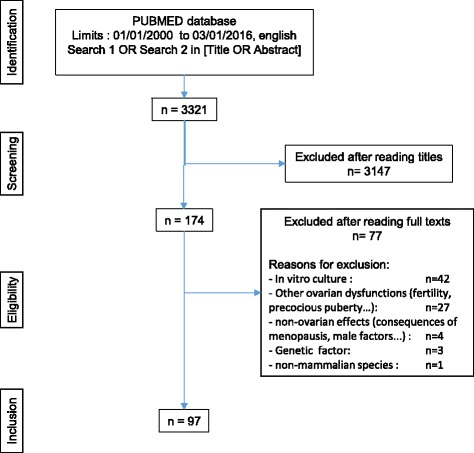
Flow chart of the selection of articles

We selected 97 citations for analysis following the flowchart presented in Fig. 2.

A cross-sectional study was conducted from 1999 to 2008 on 31,575 women, using NHANES (National Health and Nutrition Examination Survey) data. The aim was to determine the association between exposure to endocrine disruptors and age at menopause. Out of 111 analyzed EDCs, 9 polychlorinated biphenyls (PCBs), 3 pesticides, 1 furan and 2 phthalates were significantly associated with an earlier age of menopause from 1.9 to 3.8 years after adjustment for age, race/ethnicity, smoking, body mass index. A dose-response relationship was demonstrated for 14 of them, suggesting to the authors that the increase in the level of exposure to these long half-life chemicals (except phthalates) could affect ovarian function [27]. However, one has to keep in mind that nonlinear dose-responses of EDCs cannot be ignored despite some continued controversy on this point.

Finally, a review of the literature [28] focused on occupational exposure to chemicals. In total, 1074 articles were selected and 140 chemical substances were analyzed. Twenty agents were retained as likely to induce POI. Eighteen of them, acting via an increase in follicular atresia, including 3 metals (cadmium, lead and chromium), 12 synthetic organic compounds (4 pesticides, 3 solvents, 5 compounds used in industrial chemistry) and 3 belonging to the polycyclic aromatic hydrocarbons (PAHs) family.

Two synthetic organic compounds (methoxychlor and bisphenol A) acted by increasing follicular recruitment.

For 15 additional substances, data was insufficient to draw a direct link; however, these substances seem to lead to a decrease in follicle stocks [28].

Phthalates are substances that are currently used in the manufacture of plastics for a very wide spectrum of industrial applications. Two types of phthalates can be distinguished. High molecular weight phthalates (for example, di(2-ethylhexyl) phthalate [DEHP],di-isononyl phthalate [DiNP]), are used as plasticizers in the manufacture of flexible vinyl (such as polyvinyl chloride) used in consumer products (clothing, children’s toys, and household items), flooring and wall coverings, food contact applications and medical devices. They are produced in high volume because they give materials a certain flexibility and suppleness. Low molecular weight phthalates (for example, diethyl phthalate [DEP] and dibutyl phthalate [DBP]) are used in personal-care products (cosmetics), as solvents and plasticizers for cellulose acetate and in making lacquers, varnishes and coatings including the enteric coating of tablets or capsules and for controlled release formulations.

The most common one is di(2-ethylhexyl)phthalate (DEHP), and its active metabolite mono(2-ethylhexyl)phthalate (MEHP).

Human exposure to DEPH is pervasive and ubiquitous through oral ingestion, inhalation or cutaneous contact. It is estimated between 3 and 30 μg/kg/day (for review see [29]).

Phthalates are described in many studies as being endocrine disruptors altering ovarian function. The toxic effect of phthalates on the ovary rests on folliculogenesis and steroidogenesis disorders with, as a consequence, an alteration in reproductive functions, infertility and POI (for review see [30]). These substances are stable and remain in the environment for several years [29].

The literature review on phthalates and POI has allowed the study of 12 articles and 3 reviews. The study methods and primary results are described in Table 3.

**Table 3:** Effects of phthalates on ovarian function

References	Methods	Results
*Animal Data*		
Xu et al., 2010 [41]	Female rats intragastrically exposed to benzo[a]pyrene (B[a]P) 5 and 10 mg/kg, to DEHP 300 and 600 mg/kg and B[a]P + DEHP for 60 days.	⇘ number of primordial follicles (BaP 10 mg/kg ± DEHP 600 mg/kg) and of primary/secondary follicles (DEHP 600 mg/kg ± BaP 10 mg/kg) with granulosa cell apoptosis increased in DEHP, B[a]P and B[a]P + DEHP groups, implying a PPAR-mediated signaling pathway for both. No interaction effect was observed.
Moyer and Hixon, 2012 [31]	Pregnant mice exposed by oral gavage to placebo or 100, 500 or 1000 mg/kg of MEHP during gestational days 17–19.	⇘ by 1 month of reproductive lifespan in the highest F1 exposure group (9.8 ± 0.4 compared to 11.1 ± 0.6 months in the control F1 females)
Li et al., 2012 [39]	60 randomized adult mice in 4 groups exposed to 0, 125, 500 or 2000 mg/kg of DEHP by gavage for 16 weeks, 6 days/week	DEHP arrested granulosa cells in the G0/G1 phases of the cell cycle and ⇗ apoptosis in granulosa cells at 500 and 2000 mg/kg
Zhang et al., 2013 [35]	Subcutaneous injections of DEHP of 20 and 40 μg/kg in newborn mice	⇘ number of primordial follicles at puberty and adult age by accelerating follicular recruitment ⇘ DNA methylation of maternal imprinted genes (Igf2r, Peg3) in oocytes
Hannon et al., 2014 [40]	Oral exposure of adult mice to DEHP (20 μg at 750 mg/kg/day) every day for 10 or 30 days.	⇘ in the number of primordial follicles and ⇗ in the percentage of primary follicles via dysregulation of PI3K signaling
Li et al., 2014 [33]	Pregnant mice treated with DEHP from 0 to 40 μg/kg/day from 0.5 to the 18.5th day post coitum.	⇘ percentage of CpG sites in the differentially methylated regions of the oocytes in F1 mice, heritable to F2.
Zhang et al., 2015 [32]	Pregnant mice exposed to DEHP, evaluation of the ovarian reserve in offspring generations	Delay of meiosis initiation in F1 fetal ovaries with Stra8 gene hypermethylation and underexpression Acceleration of follicular recruitment in the F1 and F2 generations responsible for depletion in the primordial follicle pool.
Niermann et al., 2015 [34]	Pregnant mice exposed to DEHP from 20 μg to 750 mg/kg/day. Evaluation of the number of follicles at D8 and D21 and fertility at 3 and 9 months.	⇗ number of pre-antral follicles at D21 with a non-monotonic dose-effect 22.2% of mice exposed in utero to 20 μg/kg/day took more than 5 days to become pregnant at 3 months
Sen et al., 2015 [38]	Mice exposed to DBP at 0.01, 0.1 and 1000 mg/kg/day for 10 days.	⇘ number of antral follicles ⇗ mRNA coding for pro-apoptosis genes
Li et al., 2016 [36]	Intraperitoneal injection of DEHP in newborn mice, 20 to 40 μg/kg, every 5 days.	⇘ percentage of antral follicles. ⇗ mRNA of apoptosis genes. Accumulation of ROS.
Hannon et al., 2016 [37]	Oral exposure of adult mice to DEHP (20 or 500 mg/kg/day) every day for 10 days	⇗ BAX/BCL2 ratio in primordial follicles, ⇘ number of primordial follicles, 9 months after exposure.
*Human Data*		
Messerlian et al., 2016 [42]	Assay of phthalate metabolites in urine and antral follicle count, prospective study on 215 patients between 2004 and 2012	⇘ antral follicle count in patients whose metabolite assay was in the higher quartiles compared to lower concentrations

The collected animal data highlight different windows of susceptibility with different effects on folliculogenesis.

When considering foetal development, exposure to MEHP on pregnant mice leads to premature ovarian senescence in the F1 generation [31]. It led to a depletion of the primordial-follicle pool in the F1 and F2 generations, according to an acceleration mechanism in follicular recruitment. The authors concluded to an ovarian toxicity of phthalate with a transgenerational effect [32]. However, because they showed depletion in the primordial-follicle pool in the F1 and F2 generations, populations directly exposed to phthalates via the embryo (F1) or germ cells that it contained (F2), the effect was, more accurately, multigenerational. The effect would have been transgenerational if the F3 generation, which was the first one considered to be unexposed, had been studied and affected. This multigenerational effect is explained by the effect of phthalates on DNA methylation of imprinted genes, not only in fetal ovarian germ cells but also in the F1 and F2 offspring [33]. One study showed an increase in the number of preantral follicles in the offspring at the 21st day of life. The authors explained this increase by an acceleration in the growth of primordial and primary follicles [34].

Regarding the neonatal period, exposure of newborn mice to phthalates significantly decreased the number of primordial follicles at puberty and at the adult age by accelerating follicular recruitment [35].

When exposure to DEHP takes place during the pre-pubertal period it led to a significant reduction in the percentage of antral follicles with an increase in messenger RNA quantities of pro-apoptotic genes. DEHP causes oxidative stress and ovarian somatic cell apoptosis [36].

Finally, exposing mice during adulthood led to a significant decrease in the number of primordial [37] or antral [38] follicles according to a cell mechanism of apoptosis since there is a significant increase in messenger RNAs coding for pro-apoptotic genes [37, 39] even at low daily doses [38]. Another study found the same results with a decrease in the number of primordial follicles in addition to an increase in the percentage of primary follicles, suggesting an alteration in the beginning of folliculogenesis by accelerating primordial follicle recruitment [40]. The study on the exposure of DEHP alone or combined with tobacco showed a decrease in the number of ovarian follicles in accordance with mechanisms of apoptosis. No interaction effect was observed on the two toxicants [41].

A prospective human study on 215 patients recruited between 2004 and 2012 demonstrated a significant decrease in antral follicle count in patients whose urinary phthalate level was high compared to lower concentrations [42] after adjusting for age, BMI and smoking. However, this study was conducted on a population of infertile patients and it is difficult to generalize to the whole population.

In summary, we can conclude that phthalates disrupt ovarian function and impact the ovarian reserve by intervening at different stages of folliculogenesis [30].

Bisphenol A (BPA) is an aromatic organic compound found in plastics used for food packaging (such as polycarbonates), epoxy resins (interior coating on metal containers and beverage cans) and temperature sensitive papers (receipts). BPA is mass produced and found ubiquitously throughout the environment. Studies have shown that BPA is found in more than 90% of urine samples in a control population [43] (Table 4).

**Table 4:** Effect of bisphenol A on ovarian function

References	Methods	Results
*Animal Data*		
Rodríguez et al., 2010 [46]	Neonatal exposure of mice from D1 to D7 to BPA 0.05 or 20 mg/kg/day	⇘ percentage of primordial follicles ⇗ percentages of growing follicles at 20 mg/kg/day dose by increased recruitment, ⇗ proliferation of granulosa cells at every follicular stage
Zhang et al., 2012 [45]	Exposure of pregnant mice at 0.02, 0.04 or 0.08 mg/kg/day of orally administered BPA from Day 12.5 post coïtum.	⇘ number of primordial follicles at D3 by defaults in assembly associated with. a delay in meiosis I progression ⇘ mRNA expression of specific meiotic genes
Li et al., 2014 [47]	Intraperitoneal injection of 10, 40 or 160 mg/kg of BPA in prepubertal rats for 1 week	⇘ ovary weight, ⇘ number of primordial follicles at the highest dose, dose –dependant ⇘ in numbers of total, primary/preantral and antral follicles, ⇗ number of atretic follicles
Berger et al., 2015 [44]	Exposure of pregnant mice to BPA: 0.5, 20 or 50 μg/kg/day, histological analysis of ovaries and gene expression analysis at postnatal D4 and D21 from F1 to F3	Alteration in the number of follicles for F1, but not for F2 and F3. Alterations in ovarian gene expression at D21 with a transgenerational effect

BPA is an endocrine disruptor acting notably like an estrogen mimicker on the estrogen receptor α [17]. These effects have led French authorities to restrict the use of products containing BPA. Since July 2010, the use of baby bottles containing BPA has been prohibited. Since January 1, 2014, the manufacture and sale of any packaging containing BPA in contact with foodstuff has been prohibited.

The animal data support this argument and highlight a negative effect of BPA on ovarian reserve. In fact, exposure to BPA leads to a decrease in the number of primordial follicles regardless of the exposure window whether it is prenatal [44, 45], neonatal [46] or at the adult age [47]. In humans, a prospective study on women seeking care for infertility reported that high urinary BPA concentrations were associated with low antral follicle count after adjusting for age and BMI [48]. These data are supported by the animal and human reviews by Caserta et al. [49], Machtinger and Orvieto [50] and Richardson et al. [17] that conclude that exposure to BPA was associated with a decrease in ovarian reserve.

In utero exposure to BPA alters the ovarian reserve of the F1 generation but the results are not significant concerning the F2 and F3 generations; therefore, the negative effect of BPA does not seem to be transgenerational [44]. Nevertheless, these authors highlighted a modification in the expression of ovarian genes involved in apoptosis and steroidogenesis in later generations from F1 to F3 after in utero exposure to BPA in favor of a transgenerational effect on ovarian gene expression.

Pesticides are chemical compounds used in agriculture to fight against organisms that are considered to be harmful to crops. They are composed of different families such as insecticides, herbicides, fungicides, etc.

These organic elements have properties making them stable and lipophilic, which makes them slow to degrade over time. In this way, they remain in the environment for several years with an extensive presence in soil, food and water.

Many pesticides play the role of an endocrine disruptor in the body, altering reproductive functions and, notably, ovarian function.

The literature analysis on pesticides and POI has allowed collecting data from 6 articles and 3 reviews. The results are described in Table 5. Data was mainly on animals.

**Table 5:** Effects of pesticides on ovarian function

References	Methods	Results
*Animal Data*		
Zama and Uzumcu, 2009 [57]	Prenatal exposure of pregnant rats to MXC 20 μg or 100 mg/kg/day from embryonic D19 until postnatal day 7.	DNA hypermethylation of several ovarian genes among which ER beta. ⇗ DNA methyltransferase 3b (DNMT3b) levels in ovaries at 100 mg/kg/day
Park et al., 2014 [53]	Oral exposure of pregnant mice from gestational D12 to post-natal day 20 with 5 to 500 μg/kg dose of Simazine.	⇘ Ovarian weight and ⇗ apoptosis of granulosa cells in the F1 generation with downregulation of anti-apoptotic and proliferation genes
El-Sharkawy et al., 2014 [54]	Oral exposure of female rats to 200 mg/kg twice weekly to MXC alone, or combined with propolis (a natural anti-oxydant) 200 mg/L for 10 months	⇘ Ovarian weight, ⇗ atresia of primary, secondary and antral follicles, ⇘ ovarian antioxidant status and ⇗ in ovarian lipid peroxidation. Toxic effect neutralized using Propolis
Satar et al., 2015 [52]	Oral exposure of adult female rats to methyl parathion, every day for 8 days. Followed by ovarian histological analysis	Structural alteration of the ovarian stroma with ⇗ apoptosis phenomena in follicles during chronic exposure. = Alteration of follicular capital
Kotil and Yön, 2015 [51]	Oral exposure of adult rats to permethrine, 20 or 40 mg/kg/day for 14 days. Ovarian histological evaluation	Picnotic nucleus, condensed chromatin, alteration to the mitochondrial structure
*Human Data*		
Farr et al., 2006 [58]	Epidemiological study on 8038 women who live and work in rural American	⇗ median age at menopause by 3 months (OR = 0.87, CI 95% = 0.78–0.97) and at 5 months (OR = 0.77, CI 95% = 0.65–0.92) depending on the type of pesticides used

Alterations to cell ultrastructure, which are signs of cell apoptosis, are found in cases of exposure of female rats to permethrin, an insecticide in the pyrethroid family [51] or to methyl parathion, insecticide from the organophosphate family [52], leading to an alteration in the total number of follicles.

Exposure to Simazine [53], a herbicide from the triazine family, or to Methoxychlor (MXC) [54] an organophosphate pesticide, led to a decrease in total ovary weight, which is a sign of follicular atresia [55, 56]. Prenatal exposure to MXC in rats showed modifications to DNA methylation, which suggests epigenetic mechanisms [57].

An epidemiological research was conducted on 8038 female participants living and working on American farms. It showed that the median age at menopause was increased by 3 to 5 months, depending on the types of pesticides used [58]. After controlling for age, smoking status, and past use of oral contraceptives, pesticide use seemed to be associated with a later onset of menopause. In this study, women were exposed to multiple pesticides, which could have minimized the estimation of the effect of a specific one.

Analysis of the articles yielded results from other studies that seemed to highlight a link between exposure to pesticides and an impact on the ovarian reserve.

In one animal study, exposure of female rats to Maneb, an ethylene bisdithiocarbamate led to a reduction in the number of healthy ovarian follicles due to a dose-dependent follicular apoptosis [59].

One case-control study analyzing data from 1407 women [60], reported an earlier age at menopause for women having a higher plasma concentration of 1,1-Dichloro-2,2-bis(4-chlorophenyl)ethene, a dichlorodiphenyldichloroethylene (DDE) isomer when taking several factors into account (age, race, education, parity and lactation, physical activity, thyroid condition, body mass index and smoking).

At the same time, a cross-sectional study [61] in 219 women reported an association between elevated blood levels of specific organochlorines and an earlier age at menopause even after different confounders had been taken into account.

Cigarettes smoke contains more than 4000 chemical substances such as hydrocarbons, alcohols, phenols, aldehydes, heavy metals, etc.

The most studied components of cigarette smoke known to influence female fertility are polycyclic aromatic hydrocarbons (PAHs) which, alone, contain more than 100 chemical substances resulting from incomplete combustion. They act on the ovary via the aryl hydrocarbon receptor (AhR) present on the surface of granulosa cells. This receptor belongs to the family of transcription factors and activates the Bax gene, a pro-apoptosic gene, and the expression of cytochrome P450 that converts PAHs into even more toxic molecules [17, 62, 63]. Anderson et al. have shown that activation of this receptor could lead to a decrease in germ cells in the ovary of the human fetus [64].

All of these substances have a toxic effect on the ovary and on the reproductive function. Active cigarette smoking is associated with longer delays to conceive and with decreased results after assisted reproductive technologies [65].

We have studied the effect of cigarette smoking on POI by analyzing 22 articles, 4 reviews and one meta-analysis (Table 6). We can divide this literature into 3 categories.

**Table 6:** Effects of tobacco on ovarian function

References	Methods	Results
*Animal Data*		
Matikainen et al., 2001 [63]	Adult mouse exposure to a single intraperitoneal injection of 9,10-dimethylbenz[a]anthracene (DMBA) 50 mg/kg, a typical PAH	Activation of the aromatic hydrocarbon receptor (Ahr) driving Bax expression in oocytes, a pro-apoptosis factor
Jurisicova et al., 2007 [78]	Exposure of mice to 2 PAHs (DMBA and BaP) 3 weeks before gestation and during lactation	⇘ by one third of the ovarian follicle pool in F1 compared to unexposed offspring
Paixão et al., 2012 [79]	Exposure of 10 mice to cigarette smoke 8 h/day, 7 days/week for 15 days. Euthanized at the end of exposure or 30 days after.	Alteration in follicular growth even after discontinuing the exposure. ⇘ Number of granulosa cells in the exposed group
Kilic et al., 2012 [76]	Prospective randomized study, 25 pregnant rats exposed or unexposed to cigarette smoke.	significant ⇗ in DNA anomalies and the apoptotic index in the ovaries of exposed group ⇘ offspring ovarian reserve
Gannon et al., 2012 [77]	Mice exposed to tobacco smoke 5 day/week, for 4, 8, 9 or 17 weeks.	⇘ ovary weight and number of primordial follicles. ⇗ oxidative stress. Bcl-2 expression ⇘ but apoptosis was not induced
Sobinoff et al., 2013 [65]	Nasal exposure of mice to cigarette smoke. Ovarian toxicity analysis	⇗ Depletion of primordial and antral follicular stock via, mechanisms of apoptosis and oxidative stress
Lim et al., 2013 [89]	Mouse exposed by gavage to B[a]P at 0.2 or 10 mg/kg/d from the 7th to 16th gestational day. Effect of polymorphism of glutamate cysteine ligase	Prenatal exposure to BaP induced POI. Deletion of glutamate cysteine ligase increased the sensitivity of these ovarian effects
Camlin et al., 2016 [82]	Nasal exposure of pregnant mice to cigarette smoke for 12 weeks. Analysis of F1 ovary and oocyte quality	Abnormal proliferation of neonatal somatic cells, ⇗ apoptosis, ⇘ follicles at birth and at the adult age. ⇗ oxidative stress
*Human Data*		
Progetto Menopausa Italia Study Group, 2003 [74]	Cross-sectional study between 1997 and 1999. Inclusion of patients between ages 45 to 75 making a 1st consultation in a specialized center for menopause in Italy	No significant association between the risk of POI and smoking
Chang et al., 2007 [72]	Study of risk factors in 137 menopausal patients < 40 years, 281 between 40 and 4 years, and 1318 between 45 and 60 years	Smoking ⇗ the idiopathic risk of POI: OR = 1.82 [1.03–3.23]
Kinney et al., 2007 [110]	Effect of cigarette smoking on antral follicle count and FSH in 188 patients aged 22 to 49 years	Chronic cigarette smoking is associated with higher rates of FSH β = 0.21, 95% CI = 0.04, 0.39, but there is no significant difference on the AFC
Strohsnitter et al., 2008 [84]	Epidemiological study on 4025 women. Association between prenatal exposure to cigarette smoking and age at menopause	An association between prenatal exposure and age at menopause for patients who have never smoked (HR = 1.38, 95% CI: 1.10, 1.74). Not found in patients who smoke (HR = 1.03, 95% CI: 0.81, 1.31)
Fleming et al., 2008 [70]	Epidemiological study on 7596 women. Cotinine and FSH levels in the blood	Average age at menopause = 47.17 years for smokers compared to 48.59 years for non-smokers.
Freour et al., 2008 [81]	Analysis of 111 patients treated for infertility	Significant ⇘ of AMH levels in patients who smoke 3.06 +/− 1.68 mg/L, compared to 3.86 +/− 1.92
Lutterodt et al., 2009 [87]	Ovary analysis of 29 fetuses in the 1st trimester. Ovary analysis, compared to maternal cigarette smoking exposure	⇘ number of somatic cells (*P* ≤ 0.01). Number of oogonia not associated with prenatal exposure to maternal cigarette smoking (*P* ≤ 0.09).
Ernst et al., 2012 [86]	Prospective study on 965 pregnant women at 30 weeks amenorrhea. Evaluation of maternal cigarette smoking and study in 2008 on the fertility of their daughter (*n* = 438)	No significant association between in utero cigarette smoking exposure and the number of follicles
(Yasui et al., 2012)	Transversal study of a cohort of 24,152 Japanese nurses, research on factors associated with POI	Cigarette smoking is associated with an earlier age at menopause
Fraser et al., 2013 [83]	Analysis of a cohort of 1399 adolescents between 1991 and 2008. Study on AMH levels depending on parental exposure to cigarette smoking	Paternal, and not maternal, cigarette smoking before and during pregnancy is associated with a decrease in AMH levels.
Caserta et al., 2013 [80]	Analysis of the antral follicle count and FSH levels in 296 women, including 102 smokers	⇘ Antral follicle count and ⇗ FSH in cigarette smokers correlated to the number of pack- years.
Fowler et al., 2014 [85]	Analysis of 105 fetuses resulting from elective terminations in the 2nd trimester, 56 exposed, 49 unexposed.	Dysregulation of fetal ovarian signaling pathways
Butts et al., 2014 [88]	Survival analysis for a cohort of 410 patients. Nucleotide polymorphism study	Risk of early menopause in cigarette smokers depends on genetic polymorphism.
Tawfik et al., 2015 [71]	Cohort study of 1001 women aged 39 to 49 years. Evaluation of the association between exposure to cigarette smoke throughout the lifetime (prenatal, childhood, adulthood) and menopause status	Prenatal exposure and current cigarette smoking: 3 times more risk of early menopause (3.4; 95% CI, 1.1–10.3). Long-term exposure (>26 years) is associated to the age at menopause.
Peck et al., 2016 [73]	Analysis of primordial follicle stock in 133 patients having underwent hysterectomy for a benign pathology.	No association between follicle count and cigarette smoking
*Meta-Analysis*		
Sun et al., 2012 [66]	Analysis of 11 studies Effect of cigarette smoking on age at menopause	Dichotomous studies: OR = 0.67 (CI 95%, 0.61 to 0.73, *P* <0.01). Continuous studies: OR = −0.90 (CI 95%, −1.58 to −0.21, *P* <0.01) Cigarette smoking = independent factor in early age at menopause

Firstly, studies focusing on the relationships between cigarette smoking and age of menopause onset. According to the meta-analysis by Sun et al. [66], cigarette smoking in women is an independent factor for earlier age of menopause. Additionally, systematic reviews by Harlow and Signorello [67] and by Parente et al. [68] show an association between cigarette smoking and the age of menopause onset, but do not provide evidence that duration and quantity of exposure had any association with the age at menopause.

According to a transversal study conducted on a cohort of 24,152 Japanese nurses, cigarette smoking was a factor associated with an earlier onset of natural menopause [69].

Age at menopause is earlier from 0.8 to 1.4 years in patients who smoke according to an epidemiological study on 7596 participants [70]. In another epidemiological study, Tawfik et al. found this association for patients exposed for more than 26 years [71], and there was three times more risk for early menopause (OR: 3.4; 95% CI, 1.1–10.3). Cigarette smoking was a risk factor for POI with an odds ratio of 1.8 (1.03–3.23) [72].

One study did not show an association between age at menopause and cigarette smoking, but the authors alluded to selection and recall bias, which may have minimized the role of cigarette smoking in the onset of menopause [73]^,^[74].

Secondly, others studies focused on the relationships between cigarette smoking and follicle reserve. According to the animal review from Camlin [62], cigarette smoking has negative effects on female fertility. Exposure to tobacco induces a depletion in follicle stock [75–78], according to a mechanism of apoptosis and oxidative stress [75–78]. This depletion in follicle stock seems to persist, even in the case of discontinuing smoking [79]. Only one study highlighted an increase in oxidative stress associated with a decrease in bcl-2 (anti-apoptosis genes) without being able to show a real mechanism of cellular apoptosis (no difference in the proportion of TUNEL positive cells in exposed and non exposed ovarian follicles) [77].

Analysis of human data reinforced this theory: cigarette smoking led to a decrease in antral follicle count [80], an increase in serum FSH levels [72, 80] and a decrease in AMH (anti-mullerian hormone) levels [81] in smokers, and this seems to correlate with the number of pack-years [80]. Only the Peck et al. study [73] did not show an association between cigarette smoking and histological primordial follicle count, but the authors allude to selection bias (inclusion by surgeons, no systematic evaluation by the investigators at inclusion).

Thirdly, some studies scrutinized the effects of cigarette smoking during prenatal exposure. After in utero exposure, the Camlin et al. animal study [82] revealed a decrease in the number of follicles at birth and in adulthood by increasing pro-apoptotic phenomena. A cohort study on 1399 adolescents highlighted that paternal cigarette smoking is associated with decreased rates of AMH [83]. An association between prenatal tobacco exposure and the age at menopause is found for patients who have never smoked (HR = 1.38, 95% CI: 1.10, 1.74). However, this association is no longer found in patients who smoke (HR = 1.03, 95% CI: 0.81, 1.31) [84]. Therefore it seems that in utero exposure to cigarette smoking dysregulates fetal ovarian development signaling [85]. However, two human studies [86, 87], were unable to show a significant association between in utero cigarette smoking and the number of follicles in adulthood.

The monitoring of a cohort of 410 patients was suggestive of an interaction of genetic polymorphism with the environment. In fact, the risk of POI in cigarette smokers depended on genetic polymorphism [88]. This ovarian sensitivity, variable depending on genetic background, is shown in the murine study by Lim et al. [89], where POI induced by in utero B[a]P () exposure was increased by deletion of the glutamate cysteine ligase gene.

Phytoestrogens are plant-derived natural substances that have the particularity of mimicking the action of estrogens on their receptors (for review see [90]). The primary sources are isoflavones, of which the most common one is genistein, or lignans. An animal study has allowed studying the effect of exposure of the soy isoflavone on female rats from weaning to sexual maturity. This substance altered follicular development by increasing apoptosis of granulosa cells [91]. Neonatal exposure of mice to genistein seemed to confer early senescence of the ovarian function [92], over several generations [93] (Table 7).

**Table 7:** Effects of various pollutants on ovarian function

References	Methods	Results
Phytoestrogens		
Jefferson et al., 2007 [92]	Oral or subcutaneous exposure of neonatal mice to genistein (0.5–50 mg/kg)	Early senescence of the ovarian function, multigenerational effect, ⇗ multi-oocyte follicles from defaults in primordial follicles assembly,ovulation rates and corpora lutea at low doses and anovulation at the highest dose with arguments for disruption of the hypothalamic–pituitary–gonadal axis
Kim and Park, 2012 [94]	Review of the effect of phytoestrogens on sexual function	Disruption of ovarian function and folliculogenesis. No specification on the consequences on the ovarian reserve
Wang et al., 2014 [91]	Rats orally exposed to 50, 100 or 200 mg/kg of soy isoflavones from weaning to sexual maturity and evaluation of the ovarian reserve	Alteration of follicular development by inducing apoptosis of granulosa cells via Fas-mediated and Bcl2/Bax-mediated apoptotic pathways.
PAH		
Hombach-Klonisch et al., 2005 [96]	Review of the PAHs	Disrupts ovarian function by modulating AhR transcription
Dioxins		
Eskenazi et al., 2005 [101]	Epidemiological study conducted for 20 years on a city in Italy contaminated with TCDD following an industrial explosion. 616 patients included, impact of TCDD serum levels on age at menopause	⇗ in 6% nonsignificant risk of menopause occurring earlier with high serum concentrations of TCDD. But the trend for early menopause in the first four quintiles is statistically significant
Shi et al., 2007 [100]	Chronic exposure at low doses (0, 1, 5, 50 or 200 ng/kg/week) in female rats to TCDD from in utero life until ovarian senescence	Doses from 50 to 200 ng/kg/week: ⇗ Follicular depletion and early ovarian senescence, mediated by AhR
PCB		
Shirota et al., 2006 [104]	Oral exposure in female rats to PCB 126 at 0, 1 or 3 μg/kg/day for 2 weeks before mating until birth	⇘ ovary weight ⇗ antral follicular atresia in F1 at doses of 3 μg/kg/d. Activation of the AhR system.
Pocar et al., 2012 [105]	Exposure of mice to a mixture of PCB 0, 1, 10 or 100 mg/kg/day during pregnancy and lactation	PCB level significantly ⇗ in exposed newborns compared to the controls. Decrease in ovary weight (*p* < 0.05), ⇗ follicular atresia (*p* < 0.0001) in the F1 generation only
Perfluorinated compounds		
Knox et al., 2011 [108]	Cross-sectional study on 25,957 women	Start of menopause was earlier by several months in exposed patients
Taylor et al., 2014 [107]	Cross-sectional study of the NHANES cohort, 3011 women were studied for the association between exposure to perfluorinated compounds and the age at menopause onset	Women with elevated perfluorinated compound serum levels were younger at menopause than women with lower levels.
Alcohol		
Peck et al., 2016 [73]	Analysis of primordial follicle stock in 133 patients having underwent hysterectomy. Evaluation of behavioral habits using a questionnaire	Light to moderate alcohol consumption is associated with a higher follicle count.
Flame retardants		
Lefèvre et al., 2016 [111]	Exposure of pregnant rats to bromine flame retardants for 2 to 3 weeks	⇗ 50% in the number of antral and pre-antral follicles = Alteration of folliculogenesis
HMB = 2-hydroxy-4methoxybenzone, anti-UV protector		
Nakamura et al., 2015 [113]	Rats exposed to different doses of HMB (7–8 per group) from the 6th gestational day until the 23rd postnatal day. Effects on offspring	Delayed follicular development in the group receiving the highest dose. Exposure at less than 10,000 ppm of HMB does not seem to be associated with adverse effects on the reproductive system
Diesel		
Ogliari et al., 2013 [112]	Pregnant mice exposed daily to diesel products	⇘ Number of follicles, ⇘ ovarian reserve
2-Bromopropane		
Boekelheide et al., 2004 [109]	American review conducted in the context of the National Toxicology Program, reviewing data from five human studies	Four studies in favor of an excess risk of POI and one study without excess risk but lacks strength

A review of the literature on phytoestrogens suggested a disturbance in ovarian function and folliculogenesis without specifying the effect on the ovarian reserve [94]. However the review by Patel et al. [95] specified that genistein was responsible for a decrease in primordial, primary and secondary follicles with an increase in antral follicles. This acceleration of follicular recruitment is combined with an increase in apoptosis, and finally, follicular atresia.

Polycyclic aromatic hydrocarbons (PAH) are substances resulting from the incomplete combustion of organic fossil or non-fossil materials; one of the main components is B[a]P. This is a large family that includes certain furans, dioxins and polychlorinated biphenyls (PCB). They act by modulating the transcription of the AhR gene and have effects on reproduction [96].

Dioxins are environmental contaminants belonging to the PAH family. They mainly result from waste incineration and act via AhR, for which they are ligands. 2,3,7,8-tetrachlorodibenzo-p-dioxin (TCDD) is the most toxic member of the dioxin family [97]. It has a long half-life and accumulates in the environment and in the tissues of living organisms [98, 99].

The review of Patel et al. [95] specified that the ovary was a clear target for TCDD, altering folliculogenesis. Chronic exposure to low doses of TCDD is associated with chronic activation of AhR [96], a depletion in follicle stock that contributes to premature ovarian senescence in rats [100]. An epidemiological study from 616 Italian non menopausal women exposed to the Seveso explosion found a significant trend for an earlier age at menopause in the four first quintiles of level of exposure to TCDD at the time of explosion but not for the 5 quintiles, suggesting a nonmonotonous dose-related effect [101]. The data were adjusted for several covariates (education, parity, duration of oral contraception use).

Polychlorinated biphenyls (PCB) belong to the PAH family; there are 209 isomers resulting from complex industrial processes. They have been banned since the 1970s, but, because of their stability in the environment, they persist in adipose tissues, living organism fluids and in food (for review see [102]). They can lead to direct and indirect effects on ovary function [103]. Maternal exposure to PCB seems to have consequences on offspring, with a decrease in ovary weight in the offspring [104] and follicular atresia [105].

Perfluorinated compounds are a large family of chemical substances present in industry and everyday consumer products, notably for their anti-adhesive and anti-grease properties. They are very resistant to degradation and persist in the environment and in the food chain [106]. Two large scale epidemiological studies have demonstrated confounders-adjusted associations between exposure to perfluorinated compounds and an earlier age at menopause [107, 108].

A review led in the context of the American National Toxicology Program showed that women exposed to 2-bromopropane are at risk for the early onset of menopause [109].

Finally, other toxicants such as alcohol [73, 110], flame retardants [111], diesel [112] or anti-UV protectors [113] seem to influence the ovarian reserve but we have not found sufficient data to conclude this. Indeed, only animal studies are available for flame retardants [111], diesel [112] and anti-UV protectors [113] and discrepancies exist between the two human cross-sectional studies for alcohol [73, 110].

## Discussion

POI is a pathology in young women that can alter their fertility and, more generally, their quality of life. Its idiopathic etiology (75% of cases) could be explained by the fact that the state of ovarian reserve at a given time seems to reflect a multifactorial influence combining factors that are genetic, epidemiological and environmental in nature [114].

One of the strengths of this review may be its original nature. This is because most data available have studied the general effect of environmental pollutants on the ovary, without focusing on the mechanisms that could lead to POI. A substantial number of articles were analyzed.

The limits of this review are that most of the data have shown follicular atresia, a decrease in ovarian reserve or even an earlier age at menopause onset. Even though all of these components help create a picture of POI, they do not define POI itself. This point leads to another limit of our study that consists in the lack of systematic evaluation of the quality of evidences depicted in the studies, notably regarding the evidence for causal relationships between EDC exposures and POI. As such, our review cannot be described as a systematic review. It is noteworthy that except for tobacco smoke exposure, we couldn’t find articles reporting no association between exposure to an EDC and POI (or a witness of future POI) but this could be linked to publication bias since positive results are more likely to be published than those with negative findings (no effect of an EDC), as recently admitted by the US Endocrine Society [115].

Our review reinforces the notion that the environment, and in particular, substances acting as endocrine disruptors, seem to play a key role in the onset of mechanisms likely to cause POI. The four mechanisms leading to POI depicted in Fig. 1 can be ranked by descending order. Firstly, the induction of follicular atresia during follicular growth via an increase in oxidative stress and apoptotic phenomena (referred as situations 2 and 3 in Fig. 1). This has been shown for numerous substances: BPA [47], phthalates (DEHP [39, 41], DBP [38]), PAH [41, 96], cigarette smoke [65, 76, 77, 79, 82], pesticides [51–54], dioxins [100], genistein [91], PCB [104]. Secondly, a decreased pool of primordial follicles (situation 5 in Fig. 1) can originate from its massive atresia consecutive to exposures to PAHs [78], DEHP [37] or cigarette smoke [65, 77] or from a default in its assembly in the fetal ovary as shown for genistein [92] and BPA [45]. Thirdly; an increase in the recruitment of primordial follicles (situation 4 in Fig. 1) has been reported only for phthalates (DEHP [32, 35, 40]) and BPA [46]. Finally, we could not find any example of environmental pollutant whose exposure leads to an arrest at a particular stage of folliculogenesis (situation 6 in Fig. 1).

**Fig. 3:**
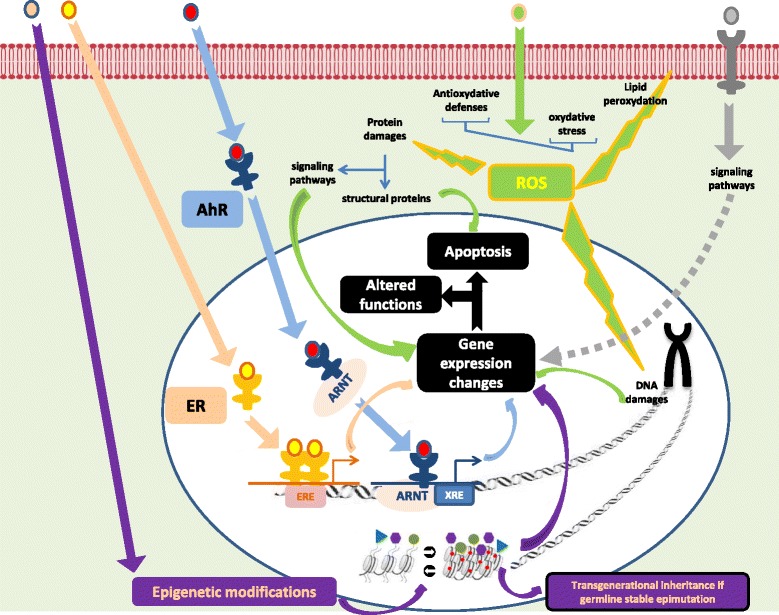
The major ways used by environmental pollutants to induce defaults in folliculogenesis leading to POI. Environmental pollutants can act through several mechanisms. • actions as endocrine disruptors as ligands to nuclear estrogen receptors (ER, *salmon arrows*) and aryl hydrocarbon receptors (AhR, *blue arrows*). Once linked to AhR, they can bind to AhR nuclear translocator (ARNT) to interfere with xenobiotoc responsive elements (XRE) from promotors and affect gene expression, notably promoting pro-apoptotic genes and inhibiting anti-apopototic genes. • creation of an imbalance between oxydative defenses and oxydative stress favoring apoptosis at different follicular stages (*green arrows*). • Modification of epigenetic marks such as DNA methylation (●) or histones post-traductional modifications (▸, , ●) affecting the transcriptional state of chromatin and therefore gene expression. • binding to several membrane receptors suchs as membrane bound ER (mER) or receptor protein tyrosine kinases (RPTK) activating the PI3 Kinase pathway are suspected (*grey dotted arrows*)

We found evidence for several molecular pathways involved in the different mechanisms leading to POI, several ones are retrieved from this review. These pathways are presented in Fig. 3. The first one encompasses an endocrine disrupting effect via the two most suspected receptors for hydrophic substances such as most EDCs: the ERs and the AhR systems. An activation of AhR system is involved in the apoptotic processes affecting follicles once activated from the primordial follicles pool due to exposure to PAH [96], dioxins [100], PCB 126 [104] or in those affecting oocytes after PAH exposure [63]. Strikingly, most of the animal studies on the ovarian consequences of exposure to EDCs known to interfere with ERs (such as phthalates, BPA, phyto-estrogens) have not demonstrated that they work via their ERs. This is probably due to the large body of evidence for such interactions reported by many in vitro studies on diverse follicular stages in several mammalian species. Since knockout animals for ER (alpha and beta) display severe ovarian phenotypes, they are useless for such demonstrations that ideally require a positive control (ex: diethylstilbestrol, ER agonist) and the absence of the EDCs effect when using a specific antagonist of the studied ER. The second molecular pathway used by environmental pollutants to influence the risk of POI resides on imbalance between oxidative defenses and the oxidative stress leading to apoptosis. Although we previously discussed the importance of those mechanisms in the altered follicular growth observed after exposure to numerous toxicants, little is known about the precise ways they are induced. Expression of typical markers of apotosis (Bax, Bcl2) or measurements of apoptosis-related cellular damages are used as witnesses of apoptosis, but without experimental data showing an excess charge of reactive oxygen species and/or insufficient antioxidant defenses. Because of the threat of a transgenerational inheritance of the defects suspected with EDCs, more detailed attention has been paid to a third mechanism by which environmental pollutants can impact folliculogenesis: epigenetics alterations. Indeed, since folliculogenesis starts during fetal development, it has been established that environmental exposure and the mother’s lifestyle can have a direct impact on the ovarian reserve of the two subsequent generations, and even beyond if epigenetic alterations affecting the germline lead to a transgenerational effect. However, although some authors showed epigenetic alterations (in term of DNA methylation exclusively) in the ovarian cells of F1 female offspring exposed to an EDC in utero [57], very few looked into F1 oocytes [32, 33], only one confirmed the alteration in F2 oocytes [33] and none looked into F3 oocytes to demonstrate the transgenerational phenotype. Furthermore, although exposure to BPA in utero leads to a transgenerational effect on ovarian gene expression in F3, folliculogenesis and estradiol secretion were normal [44]. Therefore, in the limit of our selection criteria, because altered methylation levels in F2 oocyte or altered ovarian gene expression in F3 are not indisputable proof of altered folliculogenesis in F3 ovaries, there is no evidence for an ovarian dysfunction caused by environmental pollutant with a true transgenerational inheritance. Finally, the last molecular pathway that environmental pollutants can use involves perturbations in signaling pathways known to influence folliculogenesis. For example, the phosphatidylinositol 3-kinases (PI3K) pathways are known to be regulators of primordial follicle recruitment [116]. The increased activation of primordial follicles toward primary follicles observed after adult mice exposure to DEHP has been shown to be associated with the increased expression of proteins that activate the PI3K pathway and decreased expression of genes that inhibit it [40]. Finally, apart from these different mechanisms by which EDCs could directly affect the ovaries and lead to POI, indirect mechanisms due to hypothalamo-pituitary axis dysfunctions induced by EDCs must be cited although we could not find clear data to confirm this point.

This review clearly demonstrates the lack of human data available on the subject, except for studies on cigarette smoking, which is an independent risk factor in the occurrence of POI. It also underlines the difficulty in establishing a link in humans between POI and the environment.

This difficulty could be explained by several phenomena. Firstly, exposure to a single toxicant can yield different effects depending on dose and window of exposure. Our review provides a pertinent example of this problem. In rodents, prenatal exposure to BPA affects assembly of primordial follicles in the fetal ovary [45], neonatal exposure increases the activation of follicle growth from the pool of primordial follicles [46] while prepubertal exposure increases atresia in growing follicles [47]. Secondly, human beings are subject to a complex multi-exposure to different environmental pollutants that vary over time and space making it difficult to predict the effects of one specific substance on human health [75]. This is the exposome concept [117]. Depending on these combinations, there can be a cumulative, an antagonistic or a synergy of effects that decreases the ability to detect a significant association between an exposure and the effect on POI [27]. Finally, the quantity and duration of the exposure are difficult to determine in human studies. Many substances, sometimes banned for many years, persist over the long-term and lead to chronic, ubiquitous exposure at low doses.

Beyond well identified genetic anomalies, the genetic context probably plays a role in ovarian susceptibility to environmental pollutants. However, according to some authors, arguments based on monozygotic twins cohort studies can suggest that the exposome plays a more important role than genetics in the onset of certain chronic human diseases [118].

## Conclusions

Environmental pollutants are a serious threat to human and animal reproduction with harmful effects that disturb endocrine and reproductive functions. This is a critical public health problem that needs the implementation of protection, prevention and information measures in order to fight against these environmental pollutants. One of these preventive activities may be to help health professionals to better detect patients at risk of POI in order to inform them about their reproductive ability, to limit aggravating factors and to treat them as early as possible through oocyte cryopreservation.

## Abbreviations

AhR: Aryl hydrocarbon receptor
AMH: Anti-mullerian hormone
B[a]P: benzo[a]pyrene
BPA: Bisphenol A
DDE: dichlorodiphenyldichloroethylene
DEHP: di(2-ethylhexyl)phthalate
DNA: deoxyribonucleic acid
EDCs: Endocrine disruptors
ERs: Estrogen receptors
ESHRE: European Society of Human Reproduction and Embryology
FSH: Follicle stimulating hormone
MEHP: Mono(2-ethylhexyl)phthalate
MXC: Methoxychlor
NHANES: National Health and Nutrition Examination Survey
PAH: Polycyclic aromatic hydrocarbons
PCB: Polychlorinated biphenyl
PI3K: Phosphatidylinositol 3-kinases
POI: Premature ovarian insufficiency
PRISMA: Preferred reporting items for systematic reviews and meta-analyses
RNA: Ribonucleic acid
ROS: Reactive oxygen species
